# Fibrolipoma with Osseous and Cartilaginous Metaplasia of Hoffa's Fat Pad: A Case Report

**DOI:** 10.1155/2012/547963

**Published:** 2012-08-02

**Authors:** Ioannis Gigis, Panagiotis Gigis

**Affiliations:** ^1^Orthopaedics, Aristotle University of Thessaloniki, 54124 Thessaloniki, Greece; ^2^Orthopaedic Department, Interbalkan Medical Center, Asklipiou 10, Pylaia, 57001 Thessaloniki, Greece; ^3^Anatomy, Aristotle University of Thessaloniki, 54124 Thessaloniki, Greece

## Abstract

The most common benign tumors of the mesenchyme are the lipomas. Benign fatty tumors can arise in any location in which fat is present. Fibrolipomas are characterised by fat modules. Most patients affected by such tumors are in the fifth or sixth decade of life. When very close to vital structures such as joints, they may cause functional limitations as well as pain. Osseous and chondroid metaplasia can infrequently manifest after chronic persistence. Given the rarity of this condition, a case of a big fibrolipoma of Hoffa's fat pad with osseous and cartilaginous metaplasia is reported. A 44-year-old woman presented with an enlarging soft mass on the right knee in the infrapatellar fat pad. After a thorough preoperative clinical and imaging examination, the mass was removed and sent to laboratory where the diagnosis was put. One year after surgery, both local and general condition of the patient were good and no signs of recurrence were found.

## 1. Introduction

The most common [1] benign tumors of the mesenchyme are the lipomas. It is unclear if a soft-tissue lipoma represents a benign neoplasm, a local hyperplasia of fat cells, or a combination of both processes. They may arise in any location in which fat is present, the majority found in the upper half of the body, particularly the trunk and neck, though may also develop in other sites such as the hand [2, 3]. Benign lipomatous tumors have been classified by the World Health Organisation (WHO) in the following categories: classic lipoma, lipoblastoma, lipomatosis, angiolipoma, spindle cell/pleomorphic lipoma, angiomyolipoma, myelolipoma, hibernoma, and atypical lipoma [4]. The infrapatellar Hoffa's fat pad is an intracapsular extrasynovial structure. The most common tumour or tumour-like abnormalities of the infrapatellar fat pad reported are para-articular chondroma/osteochondroma, focal pigmented villonodular synovitis, synovial lipoma, synovial chondromatosis, synovial haemangioma, ganglia/cysts, and intra-articular malignancy [5]. Some lipomas may exhibit morphological variations. These include fibrolipoma characterized by the presence of prominent bundles of mature fibrous tissue traversing the fatty lobules [6]. It can be noticed that fibrolipomas are extremely rare variants excluded from the above classifications and some times are also called benign mesenchymomas [7]. Most are subcutaneously located [8]. Uncommonly, they can manifest osseous and/or chondroid metaplasia over extended periods of time, causing additional functional problems and pain compression syndromes like the case we present here [9]. Most patients affected by lipomas are in their fifth or sixth decade of life, only rarely are children affected. Given the rarity of this condition, a case of a big fibrolipoma of Hoffa's fat pad with osseous and cartilagenous metaplasia is reported.

## 2. Case Report

**Figure 1:**
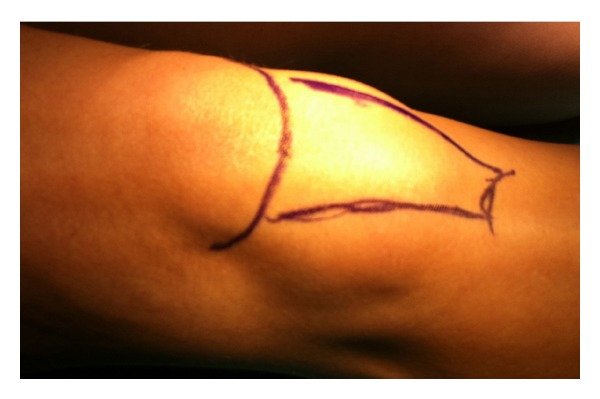
Mass observed in the infrapatellar region.

A 44-year-old woman presented with an enlarging soft mass on the right knee in infrapatellar fat pad; the mass had first been noticed 1 year ago. A gradual increase of the size of the mass had been observed by the patient during this year. Upon admission the mass could be easily seen on the right knee right below the patella in the infrapatellar region (Figure 1).

**Figure 2:**
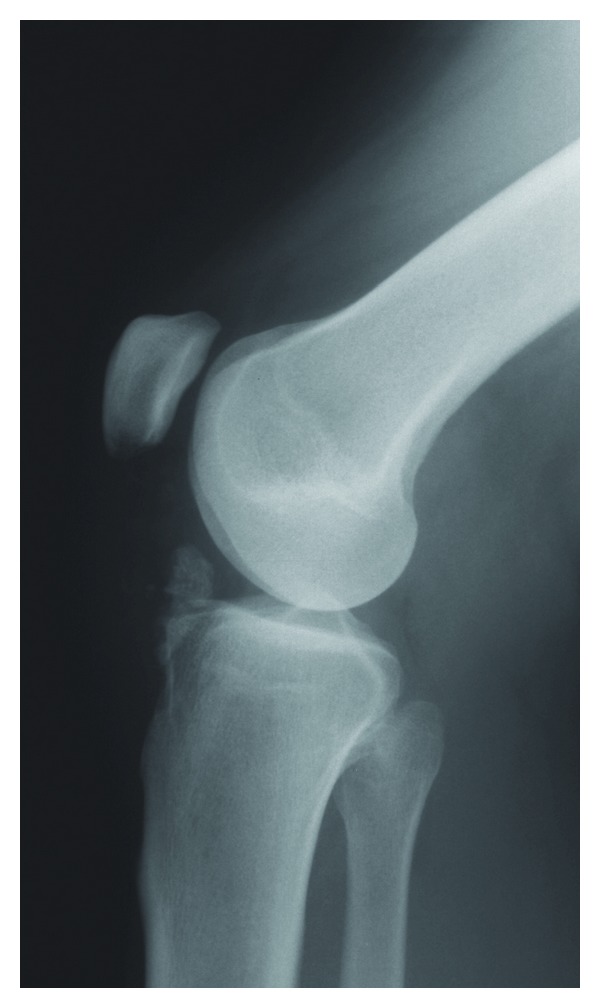
Osseous structure in the infrapatellar fat pad.

Physical examination revealed a palpable mass in Hoffa's fat pad. Flexion of the knee appeared to be limited and pain also occurred when trying to mobilize the joint. The patient complained for these limitations especially during the last few months when the mass increased in size. A standard X-ray examination revealed osseous structure in the infrapatellar site (Figure 2).

**Figure 3:**
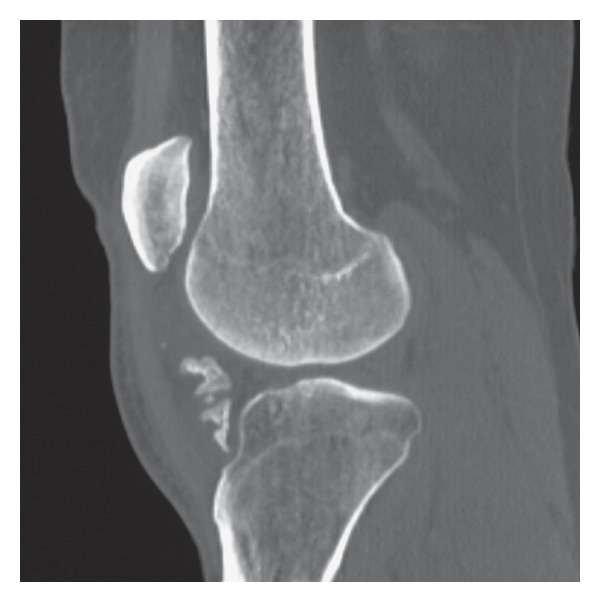
Computed tomography showing the mass with its osseous content in the infrapatellar region.

**Figure 4:**
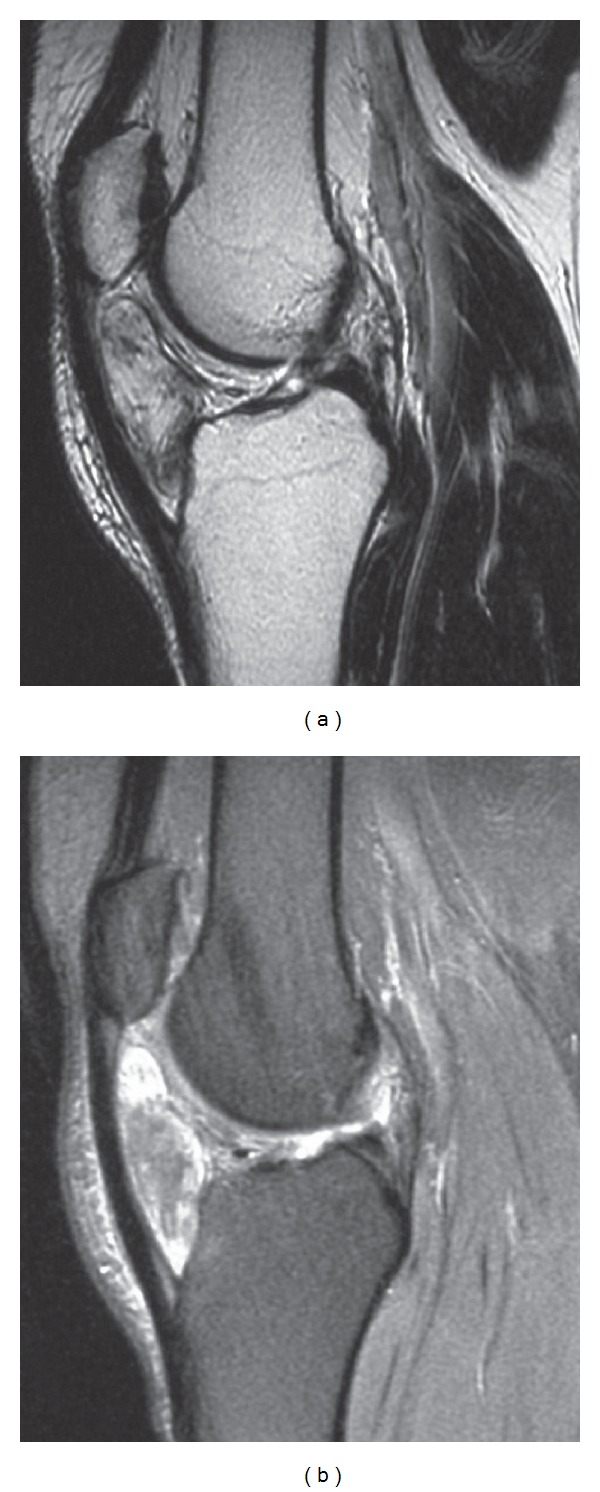
(a) Axial T1-weighted image. (b) Axial T2-weighted image showing clearly the mass in Hoffa's fat pad underlying the patellar tendon and extra-articular.

Furthermore, a CT, MRI, and a soft tissue ultrasonography were undertaken. According to the CT (Figure 3) and MRI (Figures 4(a) and 4(b)), a differential diagnosis between synovial osteochondromatosis, synovial sarcoma, and para-articular chondroma should be carried out.

**Figure 5:**
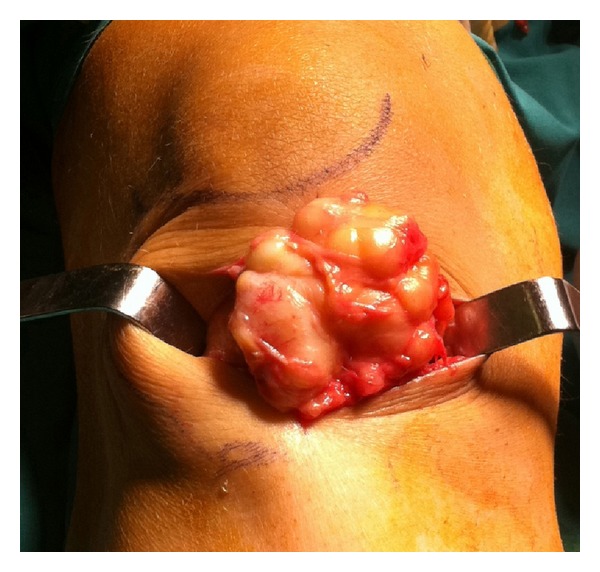
Intraoperative image showing the mass before its final excision.

**Figure 6:**
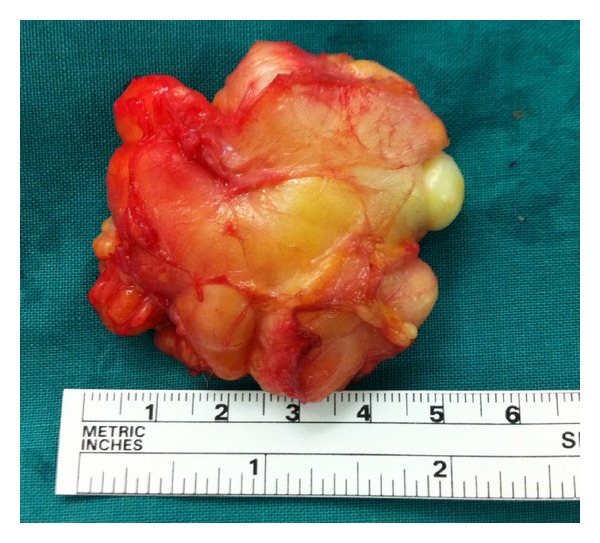
A lipomatous mass of 6 cm in diameter.

Additionally, the ultrasound showed a well-defined mature adipose tissue with the overlying patellar tendon intact. The patient was then operated and a mass of 6 cm approximately in diameter was excised under general anaesthesia along with its pedicle base (Figures 5 and 6).

**Figure 7:**
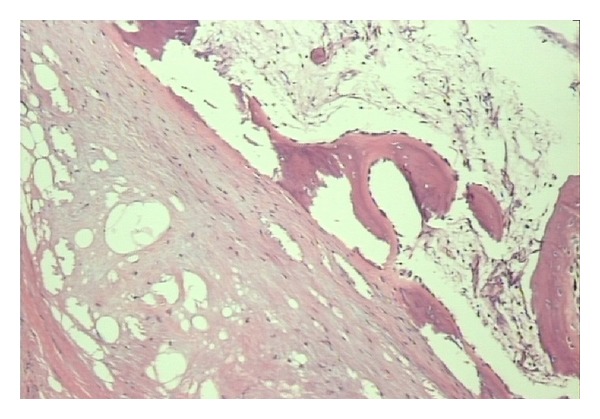
Area of osseous metaplasia.

**Figure 8:**
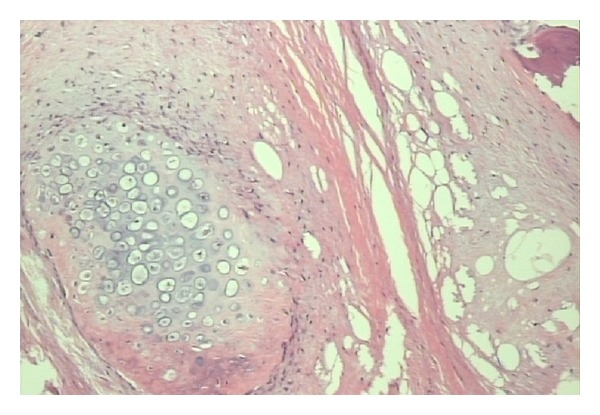
Area of cartilagenous metaplasia.

Histopathological evaluation revealed that the lesion was composed of mature adipocytes with myxoid areas as well as areas with osseous and chondroid metaplasia. The result of this histological examination suggested a fibrolipoma with osseous and cartilagenous metaplasia (Figures 7 and 8).

To date, one year after operation, both local and general condition of the patient are good, the knee has no function limitations or pain, and there are no signs of recurrence of the lesion. Followup will continue with clinical examination and imaging (radiographs and if necessary MRI or CT) annually.

## 3. Discussion

The aetiology of lipomas is unclear. They have been reported to be both sporadic and inherited [10, 11]. Lipoma cells are believed to arise from mesenchymal primordial fatty tissue cells and tend to increase in size with body weight gain [12]. When located close to vital structures such as joints, giant lipomas may cause functional limitations on account of their excessive size and weight [13] or lymphedema, pain, or nerve compression syndromes [12, 14]. For a lipoma to be referred to as “giant”, the lesion should be at least 10 cm in diameter [15]. In our case, although the lesion was only 6 cm in diameter, given the narrow space of the infrapatellar region, it caused serious limitations in knee flexion as well as pain. The malignant transformation of a lipoma into a liposarcoma is rare [16] as is the sarcomatous transformation of giant lipomas [12, 17]. It is important to differentiate giant lipomas from liposarcomas, malignant fibrous histiocytomas, and other benign soft-tissue lesions. The main concern should be the exclusion of malignancy. It has been suggested that a liposarcoma should be considered when a fatty subcutaneous tumor is more than 10 cm in diameter and has grown rapidly in recent months [18]. Fibrolipomas may develop in virtually any region that contains fat, but in general, they tend to appear on the trunk, neck, and upper extremities. Thigh has also been reported as rather uncommon region of origin [19, 20]. Never before has a fibrolipoma been reported so close to a joint, especially the knee joint. The clinical diagnosis of a fibrolipoma is established upon presentation of a nontender, semisolid mass, which has grown over extended period of time. In our case, we can only be sure that it was first noticed from the patient a year before. Radiologically, Katzer reported that soft tissue ultrasound could display mature adipose tissue embedded within fibrous mesenchymal tissue components, and even osseous metaplasia changes [9]. The ultrasound performed in our patient showed the above-mentioned mature adipose tissue but did not find the osseous metaplasia although some osseous structure was well seen in the X-ray. Computed tomography (CT) as well as magnetic resonance imaging (MRI) may be needed in such cases to rule out malignancies such as liposarcomas [19]. Indeed, both examinations, in our case, set the differential diagnosis of synovial osteochondromatosis, synovial sarcoma, and para-articular chondroma. The treatment of fibrolipomas involves surgical excision along with their well-defined pseudocapsule, followed by appropriate reconstruction. Recurrence of excised fibrolipomas has not been reported in the literature; however, there are reports of neglected cases, which eventually developed malignant transformation [7]. Dissection of these benign neoplasms is relatively straightforward.
